# Supplementary material for: Unveiling the Bioactive Potential of the Invasive Jellyfish Phyllorhiza punctata Through Integrative Transcriptomic and Proteomic Analyses
Source: Biomolecules. 2025 Aug 4;15(8):1121. doi: 10.3390/biom15081121 (PMC12383608; doi:10.3390/biom15081121)
Supplement: Supplementary file 1 [file biomolecules-15-01121-s001.zip › Suplementary Material/FileS3/PEPFOLD4/XP_065067571_14372/clusters.html]

 PEP-FOLD clusters


Download archive of all models  here   

|  |  |  |
| --- | --- | --- |
| # | file | sOPEP |
| # cluster 1 - Size: 4 |  |  |
|  | XP\_065067571\_14372-model1 | -11.0636 |
|  | XP\_065067571\_14372-model1.1 | -10.1252 |
|  | XP\_065067571\_14372-model1.2 | -9.88451 |
|  | XP\_065067571\_14372-model1.3 | -8.24338 |
| # cluster 2 - Size: 2 |  |  |
|  | XP\_065067571\_14372-model2 | -10.8829 |
|  | XP\_065067571\_14372-model2.1 | -8.03044 |
| # cluster 3 - Size: 5 |  |  |
|  | XP\_065067571\_14372-model3 | -10.7792 |
|  | XP\_065067571\_14372-model3.1 | -10.3558 |
|  | XP\_065067571\_14372-model3.2 | -10.2657 |
|  | XP\_065067571\_14372-model3.3 | -9.71321 |
|  | XP\_065067571\_14372-model3.4 | -8.68086 |
| # cluster 4 - Size: 2 |  |  |
|  | XP\_065067571\_14372-model4 | -9.71806 |
|  | XP\_065067571\_14372-model4.1 | -9.65456 |
| # cluster 5 - Size: 2 |  |  |
|  | XP\_065067571\_14372-model5 | -9.51586 |
|  | XP\_065067571\_14372-model5.1 | -7.74255 |
| # cluster 6 - Size: 1 |  |  |
|  | XP\_065067571\_14372-model6 | -9.20709 |
| # cluster 7 - Size: 2 |  |  |
|  | XP\_065067571\_14372-model7 | -9.0823 |
|  | XP\_065067571\_14372-model7.1 | -7.45932 |
| # cluster 8 - Size: 1 |  |  |
|  | XP\_065067571\_14372-model8 | -8.82029 |
| # cluster 9 - Size: 2 |  |  |
|  | XP\_065067571\_14372-model9 | -8.47131 |
|  | XP\_065067571\_14372-model9.1 | -8.46357 |
| # cluster 10 - Size: 2 |  |  |
|  | XP\_065067571\_14372-model10 | -8.46043 |
|  | XP\_065067571\_14372-model10.1 | -8.03484 |
| # cluster 11 - Size: 1 |  |  |
|  | XP\_065067571\_14372-model11 | -8.31028 |
| # cluster 12 - Size: 2 |  |  |
|  | XP\_065067571\_14372-model12 | -8.12899 |
|  | XP\_065067571\_14372-model12.1 | -7.00927 |
| # cluster 13 - Size: 1 |  |  |
|  | XP\_065067571\_14372-model13 | -8.09076 |
| # cluster 14 - Size: 2 |  |  |
|  | XP\_065067571\_14372-model14 | -7.92775 |
|  | XP\_065067571\_14372-model14.1 | -7.47044 |
| # cluster 15 - Size: 8 |  |  |
|  | XP\_065067571\_14372-model15 | -7.88838 |
|  | XP\_065067571\_14372-model15.1 | -6.202 |
|  | XP\_065067571\_14372-model15.2 | -6.00514 |
|  | XP\_065067571\_14372-model15.3 | -5.96723 |
|  | XP\_065067571\_14372-model15.4 | -5.89169 |
|  | XP\_065067571\_14372-model15.5 | -5.71377 |
|  | XP\_065067571\_14372-model15.6 | -5.70074 |
|  | XP\_065067571\_14372-model15.7 | -5.41464 |
| # cluster 16 - Size: 3 |  |  |
|  | XP\_065067571\_14372-model16 | -7.79226 |
|  | XP\_065067571\_14372-model16.1 | -7.64852 |
|  | XP\_065067571\_14372-model16.2 | -7.62345 |
| # cluster 17 - Size: 7 |  |  |
|  | XP\_065067571\_14372-model17 | -7.65997 |
|  | XP\_065067571\_14372-model17.1 | -7.44469 |
|  | XP\_065067571\_14372-model17.2 | -7.2414 |
|  | XP\_065067571\_14372-model17.3 | -5.90325 |
|  | XP\_065067571\_14372-model17.4 | -5.84343 |
|  | XP\_065067571\_14372-model17.5 | -5.79895 |
|  | XP\_065067571\_14372-model17.6 | -5.74518 |
| # cluster 18 - Size: 4 |  |  |
|  | XP\_065067571\_14372-model18 | -7.58829 |
|  | XP\_065067571\_14372-model18.1 | -7.53728 |
|  | XP\_065067571\_14372-model18.2 | -7.49569 |
|  | XP\_065067571\_14372-model18.3 | -7.24192 |
| # cluster 19 - Size: 1 |  |  |
|  | XP\_065067571\_14372-model19 | -7.5718 |
| # cluster 20 - Size: 2 |  |  |
|  | XP\_065067571\_14372-model20 | -7.45844 |
|  | XP\_065067571\_14372-model20.1 | -6.73271 |
| # cluster 21 - Size: 4 |  |  |
|  | XP\_065067571\_14372-model21 | -7.38768 |
|  | XP\_065067571\_14372-model21.1 | -7.11551 |
|  | XP\_065067571\_14372-model21.2 | -5.98999 |
|  | XP\_065067571\_14372-model21.3 | -5.90376 |
| # cluster 22 - Size: 2 |  |  |
|  | XP\_065067571\_14372-model22 | -7.3442 |
|  | XP\_065067571\_14372-model22.1 | -5.8657 |
| # cluster 23 - Size: 2 |  |  |
|  | XP\_065067571\_14372-model23 | -7.26497 |
|  | XP\_065067571\_14372-model23.1 | -6.22026 |
| # cluster 24 - Size: 3 |  |  |
|  | XP\_065067571\_14372-model24 | -7.24939 |
|  | XP\_065067571\_14372-model24.1 | -6.01769 |
|  | XP\_065067571\_14372-model24.2 | -5.69014 |
| # cluster 25 - Size: 1 |  |  |
|  | XP\_065067571\_14372-model25 | -7.14012 |
| # cluster 26 - Size: 1 |  |  |
|  | XP\_065067571\_14372-model26 | -6.93551 |
| # cluster 27 - Size: 1 |  |  |
|  | XP\_065067571\_14372-model27 | -6.85774 |
| # cluster 28 - Size: 2 |  |  |
|  | XP\_065067571\_14372-model28 | -6.79289 |
|  | XP\_065067571\_14372-model28.1 | -5.84274 |
| # cluster 29 - Size: 1 |  |  |
|  | XP\_065067571\_14372-model29 | -6.71752 |
| # cluster 30 - Size: 1 |  |  |
|  | XP\_065067571\_14372-model30 | -6.6614 |
| # cluster 31 - Size: 2 |  |  |
|  | XP\_065067571\_14372-model31 | -6.45368 |
|  | XP\_065067571\_14372-model31.1 | -6.22527 |
| # cluster 32 - Size: 1 |  |  |
|  | XP\_065067571\_14372-model32 | -6.37352 |
| # cluster 33 - Size: 2 |  |  |
|  | XP\_065067571\_14372-model33 | -6.35451 |
|  | XP\_065067571\_14372-model33.1 | -5.86023 |
| # cluster 34 - Size: 2 |  |  |
|  | XP\_065067571\_14372-model34 | -6.19543 |
|  | XP\_065067571\_14372-model34.1 | -5.89255 |
| # cluster 35 - Size: 2 |  |  |
|  | XP\_065067571\_14372-model35 | -6.19522 |
|  | XP\_065067571\_14372-model35.1 | -6.00931 |
| # cluster 36 - Size: 1 |  |  |
|  | XP\_065067571\_14372-model36 | -6.09767 |
| # cluster 37 - Size: 1 |  |  |
|  | XP\_065067571\_14372-model37 | -6.05612 |
| # cluster 38 - Size: 4 |  |  |
|  | XP\_065067571\_14372-model38 | -6.02855 |
|  | XP\_065067571\_14372-model38.1 | -5.97191 |
|  | XP\_065067571\_14372-model38.2 | -5.72753 |
|  | XP\_065067571\_14372-model38.3 | -5.66669 |
| # cluster 39 - Size: 1 |  |  |
|  | XP\_065067571\_14372-model39 | -5.99096 |
| # cluster 40 - Size: 2 |  |  |
|  | XP\_065067571\_14372-model40 | -5.98342 |
|  | XP\_065067571\_14372-model40.1 | -5.4487 |
| # cluster 41 - Size: 3 |  |  |
|  | XP\_065067571\_14372-model41 | -5.958 |
|  | XP\_065067571\_14372-model41.1 | -5.90043 |
|  | XP\_065067571\_14372-model41.2 | -5.68149 |
| # cluster 42 - Size: 1 |  |  |
|  | XP\_065067571\_14372-model42 | -5.85535 |
| # cluster 43 - Size: 1 |  |  |
|  | XP\_065067571\_14372-model43 | -5.81428 |
| # cluster 44 - Size: 1 |  |  |
|  | XP\_065067571\_14372-model44 | -5.71432 |
| # cluster 45 - Size: 1 |  |  |
|  | XP\_065067571\_14372-model45 | -5.70911 |
| # cluster 46 - Size: 1 |  |  |
|  | XP\_065067571\_14372-model46 | -5.6669 |
| # cluster 47 - Size: 1 |  |  |
|  | XP\_065067571\_14372-model47 | -5.64378 |
| # cluster 48 - Size: 1 |  |  |
|  | XP\_065067571\_14372-model48 | -5.60003 |
|  |  |  |
